# *Enterococcus faecalis* strains derived from wild bird provide protection against *Clostridium perfringens* challenge in locally-sourced broilers

**DOI:** 10.3389/fvets.2025.1601605

**Published:** 2025-05-15

**Authors:** Gerui Zhang, Ainul Zahra, Tianrui Yang, Qiyu Guo, Yan Sun, Yuhang Zhang, Yupeng Gao, Yuxi Zhang, Mingyue Wang, Jingshuo Gong, Haibin Huang, Zhannan Wang, Chunfeng Wang, Yanlong Jiang

**Affiliations:** College of Animal Medicine, Jilin Provincial Engineering Research Center of Animal Probiotics, Jilin Provincial Key Laboratory of Animal Microecology and Healthy Breeding, Engineering Research Center of Microecological Vaccines (Drugs) for Major Animal Diseases, Ministry of Education, Jilin Agricultural University, Changchun, China

**Keywords:** strain screening, probiotic, antibiotic alternatives, *Clostridium perfringens*, *Enterococcus faecalis*

## Abstract

**Introduction:**

Necrotic enteritis (NE), caused by *Clostridium perfringens*, has seen a surge in chicken populations recently due to the ban on antibiotic growth promoters in feed.

**Methods:**

In this research, screening and identification of probiotics with strong antagonistic ability against *C. perfringens* from 34 wild bird fecal isolates, followed by analysis of probiotic characteristics and carbon source metabolic activity. Strains exhibiting favorable antagonistic activity against *C. perfringens* were subsequently employed in vivo study to evaluate their protective efficacy against *C. perfringens* challenge in locally-sourced broilers.

**Results:**

The results showed that *Enterococcus faecalis* strains YL-EF25 and YL-EF32 were selected based on their ability to inhibit the growth and biofilm formation of *C. perfringens*. These two strains demonstrated good tolerance to bile salts, artificial gastric juice, and phenol, as well as metabolic activity toward dietary fiber and propionic acid precursor substances. *In vivo* tests on locally-sourced broilers revealed that NE induced body weight loss, intestinal lesions, and intestinal inflammation, as well as imbalance in the gut microflora. Administration of *E. faecalis* YL-EF25 and YL-EF32 can alleviate these symptoms. We find that feed supplementation with YL-EF25 and YL-EF32 reduced the lesion score of challenged chicks (*p* < 0.05), with increased tight junction-related gene expression (Occludin and ZO-1) and decreased proinflammatory cytokine (TNF-α and IFN-γ) expression in jejunum compared with NE-induced broilers (*p* < 0.05). Furthermore, *E. faecalis* YL-EF25 can boost peripheral blood lymphocyte proliferation activity (*p* < 0.05).

**Discussion:**

These finding indicated that addition of *E. faecalis* YL-EF25 and YL-EF32 improved growth performance and mitigated NE-induced gut injury, possibly by strengthening intestinal mucosal barrier function and restoring effects on the ileal microbial composition in *C. perfringens-challenged* broilers.

## 1 Introduction

Necrotic enteritis (NE) is an acute infectious disease caused by *Clostridium perfringens*. As a Gram-positive anaerobic bacterium, *C. perfringens* is widely distributed in natural environments and animal intestines, where it poses a latent threat. Under conditions of intestinal dysbiosis, *C. perfringens* produces toxins that disrupt the intestinal barrier, ultimately leading to the clinical manifestations of NE (1, 2). In poultry industry, the incorporation of antibiotic growth promoters (AGPs) into feed has proven to be a potent strategy for mitigating the occurrence of NE outbreaks (3). However, due to concerns about the spread of bacterial resistance resulting from the overuse of AGPs, many European countries have banned their use in feed, and in 2020, China also announced the prohibition of 11 antibiotics as feed additives (4). Nevertheless, the ban on these antibiotics in poultry production has led to a series of issues, including outbreaks of bacterial infectious diseases and decreased production profits (5). Currently, NE in broilers has become one of the significant factors affecting the development of the poultry industry, causing severe economic losses of approximately $6 billion annually. Therefore, there is an urgent need to find alternatives to AGPs for controlling the occurrence of NE (6). Studies have identified several products with potential as AGP alternatives, such as probiotics (7), antimicrobial peptides (8) and plant extracts (9). Additionally, the development of vaccines targeting toxins secreted by *C. perfringens*, such as the alpha toxin and pore-forming toxin NetB (10), represents another promising approach.

*Enterococcus faecalis*, a Gram-positive, facultatively anaerobic bacterium, constitutes an essential component of the normal intestinal microbiota in animals. *E. faecalis* has tolerance to various harsh environments, including elevated bile salts and acidity, this organism demonstrates a minimal nutritional requirement for growth and is notably facile to cultivate (11, 12). In animal husbandry, *E. faecalis* usually plays a positive role. In 1984, both the US FDA and AAFCO allowed *E. faecalis* to be used as a safe microbial feed additive. In 2013, the Chinese Ministry of Agriculture approved the use of *E. faecalis* as a feed additive in farmed animals. Probiotics are defined by the WHO as live microorganisms that, when administered in adequate amounts, confer a health benefit on the host (13). As previous studies have shown, probiotics play a leading role in the intestinal homeostasis balance, including maintaining intestinal microbiota stability, inhibiting the colonization of harmful bacteria, enhancing intestinal barrier function, and regulating inflammatory and immune responses (14). Additionally, probiotics can inhibit the invasion of pathogens by producing antimicrobial substances such as bacteriocins and organic acids or through competitive inhibition, thereby reducing the incidence of gastrointestinal diseases (15). Wu et al. demonstrated that the addition of *Enterococcus faecium* NCIMB 11181 to the diet effectively reduced the damage caused by *C. perfringens*-induced NE in broilers by enhancing the expression of intestinal tight junction protein CLDN-1 and heat shock protein HSP70, upregulating intestinal gene expression of Toll like receptor negative regulator PI3K and growth factor GLP-2 and regulating intestinal microbiota composition (16). García-Vela et al. identified *Enterococcus faecium* X2893 and X2906 (ST722) from poultry sources as promising anti-*Clostridium perfringens* candidates, exhibiting bacteriocin activity (enterocin A/B), absence of acquired antibiotic resistance genes/plasmids, and colonization-associated acm gene expression, suggesting their potential as antibiotic-alternative protective cultures in poultry farming (17).

In the present study, we isolated and screened the wild bird-derived *E. faecalis* strains YL-EF25 and YL-EF32 with potential probiotic effects and antagonistic activity against *C. perfringens*. To demonstrate that YL-EF25 and YL-EF32 can provide protection against *C. perfringens* challenge in broiler chickens, we further investigated the effects of *E. faecalis* YL-EF25 and YL-EF32 on growth performance, immune responses, intestinal barrier function, intestinal inflammatory injury and intestinal microbiota, in order to prevent and control *C. perfringens* infection.

## 2 Materials and methods

### 2.1 Pathogenic bacteria and medium

*Clostridium perfringens* strain CP4 was obtained from D.A. Barnum, Department of Pathobiology, University of Guelph (18). Trypticase Sulfite Neomycin Agar (TSN) and Fluid Thioglycollate medium (FT) were used for culturing *C. perfringens*. Tryptic Soy Broth medium (TSB) was used for biofilm assay of *C. perfringens*. De Man, Rogosa and Sharpe medium (MRS) was used for screening and cultivating lactic acid bacteria. Medium and reagents were purchased from Qingdao Hi-Tech Industrial Park Hope Bio-Technology Co., Ltd. (Qingdao, China), and Solarbio Science & Technology Co., Ltd. (Beijing, China).

### 2.2 Isolation and identification of lactic acid bacteria

Fecal samples were collected from healthy wild birds in a natural scenic area in Jilin Province, China. The samples were serially diluted to 10^−7^ and spread onto MRS agar plates, which were then incubated at 37°C under aerobic conditions for 24 h. Bacteria colonies were selected and streaked onto fresh MRS agar plates for further activation. The activated colonies were then inoculated into 5 mL MRS broth and cultivated at 37°C for 24 h.

### 2.3 Screening of isolated strains for strong inhibitory activity against *Clostridium perfringens*

The agar well diffusion method, with some modifications based on the previous description (19), was employed to evaluate the antibacterial activity. Briefly, *C. perfringens* was used as the indicator bacterium. Both the isolated strains and the indicator bacterium were separately inoculated into MRS broth and FT medium, respectively, and cultivated at 37°C under anaerobic conditions for 16 h to reach the stationary phase. The indicator bacterium was then adjusted to an OD_600_ of 0.8 (around 1 × 10^8^ CFU/mL). The isolated strains were centrifuged at 4000 rpm for 5 min to harvest the supernatant, which was subsequently filtered through a 0.22 μm membrane to obtain sterile cell-free supernatant (CFS). The adjusted indicator bacterium was evenly spread onto TSN agar plates, and place an Oxford cups on a TSN agar plates, the Oxford cups were filled with the CFS. After incubation at 37°C under anaerobic conditions, the diameters of the inhibition zones were recorded. The strains exhibiting the significant antibacterial activity was selected for subsequent experiments.

### 2.4 PCR amplification and DNA sequencing of 16S rRNA

The top 4 strains with the high antibacterial activity were selected and genomic DNA were extracted using an Ezup column-based bacterial genomic DNA extraction kit (Sangon Biotech Co., Ltd., Shanghai, China), with the procedure performed according to the manufacturer’s instructions. PCR of the 16S rDNA with primers 27F (5′-AGAGTTTGATCCTGGCTCAG-3′) and 1492R (5′-TACGGCTACCTTGTTACGACTT-3′) (20) used 0.5 μL of DNA template (20–50 ng/μL), 2.5 μL of 10 × PCR buffer (with 50 mol/L Mg^2+^), 1.0 μL of dNTP (2.5 mmol/L each), 0.2 μL of DreamTaq™ DNA Polymerase (5 U/μL) (Thermo Scientific, Waltham, MA, USA), and 0.5 μL of primers (10 μmol/L each), and ddH2O was added to achieve a final volume of 25 μL. PCR was performed on a 2720 thermal cycler (Applied Biosystems, Foster City, CA, USA) based on the following procedure: 5 min of denaturation at 95°C, 30 cycles of denaturation at 94°C for 30 s, annealing at 57°C for 30 s, extension at 72°C for 90 s, and a final extension at 72°C for 10 min. The amplified fragments were sequenced using a 3730XL sequencer (Applied Biosystems) by Sangon. The nucleotide sequences were aligned with the available sequences in the GenBank database through NCBI blasting,^1^ and a phylogenetic tree was constructed using MEGA software (version 11) (21) in order to determine the species of the bacterial strains.

### 2.5 Hemolytic activity, growth curve, and acid production curve

The 4 strains of *E. faecalis* with the strongest antagonistic activity against *C. perfringens*, designated as YL-EF7, YL-EF25, YL-EF28, and YL-EF32, were streaked onto plates containing 5% sheep blood agar medium and incubated at 37°C to observe their hemolytic ability. Additionally, these strains were inoculated into 100 mL of MRS broth and cultivated at 37°C with shaking at 120 rpm for 36 h. Samples of 5 mL were taken at the 2nd, 4th, 6th, and every 6th hour thereafter to measure the OD_600_ value and pH of the bacterial suspension. The data were recorded and used to plot growth curves and acid production curves.

### 2.6 Tolerance to artificial gastric acid and bile salts

The 4 strains were streaked onto MRS agar plates and incubated at 37°C. Colonies were then picked and inoculated into MRS broth, which was cultivated at 37°C overnight. The bacterial suspension concentration was adjusted to 1 × 10^8^ CFU/mL. To determine the initial bacterial count, 1 mL of the adjusted suspension was serially diluted in PBS and spread onto MRS agar plates, which were then incubated at 37°C for 24 h to calculate the initial bacterial concentration [log_10_ CFU/mL (I)]. The remaining suspension was centrifuged at 5000 rpm for 10 min, and the bacterial pellet was resuspended in corresponding bile salt solutions (0.2, 0.25%) and artificial gastric acid solution (0.32% pepsin, pH = 2) prepared in PBS. The suspensions were then incubated at 37°C for 3 h. After incubation, the suspensions were serially diluted in PBS and spread onto MRS agar plates, which were incubated at 37°C for 24 h to calculate the bacterial count in the treated groups [log_10_ CFU/mL (L)] and the survival rate. The survival rate was calculated using the following formula:


$$\text{Survival rate}\;(\%)=\frac{\log10\;\mathrm{CFU}/\mathrm{mL}(L)}{\log10\;\mathrm{CFU}/\mathrm{mL}(I)}\times 100$$

### 2.7 Phenol tolerance

The phenol tolerance test for *E. faecalis* was conducted based on the previous description method (22), with some modifications. The overnight cultures of the *E. faecalis* strains were inoculated into MRS broth containing different concentrations of phenol (0.1, 0.2, 0.3, and 0.4%) and cultivated at 37°C for 24 h. The OD_600_ values of the bacterial suspensions in each group were measured.

### 2.8 Biofilm formation

The biofilm inhibition test was performed according to the previous described method (23), with some modifications. Overnight cultures of *C. perfringens* (CP) and *E. faecalis* (YL-EF7/25/28/32) were washed once with PBS and resuspended in TSB medium to an OD_600_ of 0.1. A 100 μL aliquot of the CP suspension was inoculated into each well of a 96-well polystyrene tissue culture plate, followed by the addition of 100 μL or 200 μL of the EF suspension or filter-sterilized CFS. Each well was supplemented with TSB medium to a total volume of 300 μL per well. Control groups included CP alone (300 μL CP) and a negative control (300 μL TSB). The experimental groups were designed as follows: CP, CP + EF, CP + TSB + EF, CP + CFS, and CP + CFS + TSB. The cultures were incubated in sealed culture plate to prevent evaporation and incubate at 25°C for 5 days. The OD_600_ values of the supernatants from each group were measured, and the viable counts of CP in the planktonic phase were determined using selective medium (TSN). The wells containing biofilms were gently washed twice with PBS, and 300 μL of a 0.1% crystal violet solution was added to each well and stained at room temperature for 30 min. The crystal violet solution was carefully removed, and the wells were washed twice more with PBS. The biofilm-bound crystal violet was extracted with 300 μL of methanol for 30 min, and the methanol solution was transferred to a new 96-well plate. The OD570 values were measured using a multifunctional microplate reader (TECAN, Switzerland). The ratio of A570 to OD_600_ was used as a relative measure of biofilm production.

### 2.9 Phenotypic analysis of carbon source utilization

The 71 carbon sources utilization and sensitivity to 23 chemicals of *E. faecalis* YL-EF25 and YL-EF32 were assessed using Biolog GEN III microplates (Biolog, USA) to provide a phenotypic profile of their carbon source metabolism. Briefly, the strains were streaked onto MRS agar medium for activation and then inoculated into special inoculating fluid (IF) to prepare bacterial suspensions with the recommended optical density. A 100 μL aliquot of each suspension was inoculated into a GEN III microplate and incubated in an OmniLog instrument for 30 h. During incubation, bacterial respiration reduces tetrazolium redox dye, turning it purple. The OmniLog automated microbial identification system automatically collects phenotypic data.

### 2.10 Animal experimental design

The animal experiments were conducted on 90 one-day-old broilers purchased from a poultry farm in Changchun, China, and all animal experiments were approved by the Ethics Committee for Animal Protection and Use of Jilin Agricultural University. The rearing temperature was controlled at 33°C for the first 7 days, and then decreasing cumulatively 3°C in every week until the temperature of 24°C was reached. The humidity was maintained at around 60%, and the animal room was illuminated for 24 h during the first 3 days, followed by 20 h of light per day. The animals were allowed free access to water and feed. A total of 90 male broiler chickens were randomly divided into 5 treatments groups, with 6 cages in each group and 3 chickens per cage.

**Figure 1:**
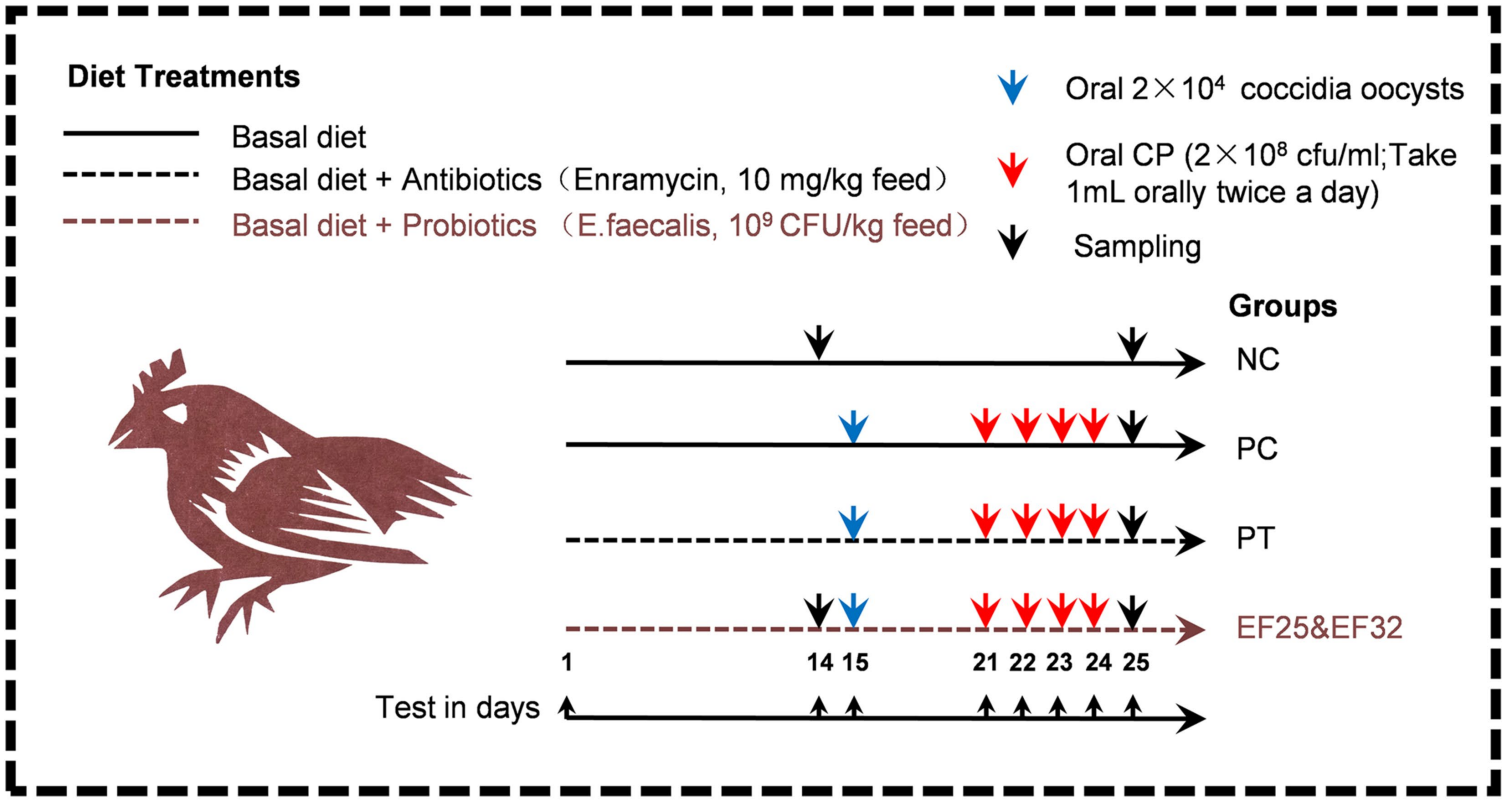
Schematic diagram of the animal experiment design for the prevention and control of necrotic enteritis using *E. faecalis* YL-EF25 and YL-EF32. The method of adding antibiotics and probiotics is to thoroughly mix and stir the additives into the feed. During the necrotic enteritis modeling stage (D15-D25), adding fish meal to the basal diet of necrotic enteritis modeling stages (PC, PT, EF25, EF32) increased the crude protein content of the diet to 28%. On the 15th day, 20,000 coccidian oocysts were orally inoculated. From the 21st to the 24th day, fresh CP bacterial solution was orally inoculated twice a day. On the 25th day, sampling.

The treatments were as follows: (1) negative control group birds fed a basal diet and not challenged (NC); (2) CP-infected group birds fed a basal diet and orally challenged with 2 × 10^4^ coccidia (*Eimeria tenella*) oocysts and 4 × 10^8^ cfu CP (PC); (3) antibiotic group fed a basal diet containing 10 mg of the Enramycin/kg of feed and then orally challenged with 2 × 10^4^ coccidia oocysts and 4 × 10^8^ cfu CP (PT); (4) *E. faecalis* YL-EF25 group fed a basal diet containing *E. faecalis* YL-EF25 (10^9^ cfu/kg of feed) and then orally challenged with 2 × 10^4^ coccidia oocysts and 4 × 10^8^ cfu CP (EF25); and (5) *E. faecalis* YL-EF32 group fed a basal diet containing *E. faecalis* YL-EF32 (10^9^ cfu/kg of feed) and then orally challenged with 2 × 10^4^ coccidia oocysts and 4 × 10^8^ cfu CP (EF32). The basal diets were formulated according to the Chinese Feeding Standard for Chickens. The nutrient ingredient compositions of the feed are shown in Supplementary Table S1. From the 15th to the 25th day, adjust the crude protein content in the diet to 28% using fish meal to increase the effect of CP infection. The specific details of the animal experimental design are shown in Figure 1. The body weights of the chickens were measured on days 0, 14, and the final day (day 25) of the experiment. Feed intake (FI), body weight gain (BWG), and feed conversion ratio (FCR) were calculated and recorded for further analysis. The FCR value was calculated using the following formula:


$$\mathrm{FCR}(g/g)=\frac{\mathrm{FI}(g)}{\mathrm{BWG}(g)}$$

### 2.11 Lymphocyte proliferation

Peripheral blood lymphocytes were isolated from chickens using a lymphocyte separation kit (Solarbio, China). On day 14, lymphocytes (1 × 10^6^ cells/well in 96-well plates) were stimulated with 20 μg/mL Concanavalin A for 48 h. Proliferative activity was then quantified using a CCK-8 assay (Melunbio, China), with the stimulation index (SI) calculated according to the manufacturer’s protocol.

### 2.12 Lesion score

On day 25, the lesion scores of the small intestine were evaluated. Chickens from each group were euthanized, and their small intestines were dissected for objective lesion scoring. The scoring details were based on the method described by Shojadoost et al., where intestinal lesions were rated on a scale of 0 to 5 (24).

### 2.13 Extraction of total RNA from jejunum and real-time quantitative PCR

Jejunum samples were collected from broiler chickens on day 25. Total RNA was isolated using TRIzol reagent (Transgen, China) and quantified. Subsequently, cDNA synthesis was performed using the BeyoRT™ II cDNA First Strand Synthesis Kit (RNase H-) (Beyotime, China). The expression levels of the respective genes were detected using BlasTaq™ 2 × qPCR MasterMix (Beyotime, China) and quantified on an Applied Biosystems 7500 Instrument. GAPDH was utilized as an endogenous control in the comparative CT method. The primers used are listed in the Supplementary Table S2.

### 2.14 Ileal microbiome

Total genomic DNA from ileum samples was extracted using the E.Z.N.A™ Mag-Bind Soil DNA Kit (Omega, USA) according to the manufacturer’s instructions. On Day 26, broiler ileal digesta were sampled. DNA was extracted from ileal digesta using the E.Z.N.A™ Mag-Bind Soil DNA Kit (Omega, USA) according to the guidelines. According to the specifications outlined by Illumina, all DNA samples were pretreated for MiSeq compositional sequencing. The V3-V4 region of the 16S rRNA gene was amplified, and Illumina index primers were attached in two separate PCRs. FLASH software (v1.2.7) was used to generate raw tags (25). Effective tags were obtained by the UCHIME algorithm (26) and QIIME (v1.7.0) analysis (27) UPARSE software (v7.0.1001) was used to analyze sequences, and the sequences were clustered at 97% similarity as operational taxa (OTUs). The GreenGene database was used to compare sequences and classify the different classification levels of these sequences. Microbial diversity was detected through QIIME software and Python scripts.

### 2.15 Statistical analysis

All of these data were analyzed with SPSS Version 20.0 for Windows (SPSS Inc., Chicago, IL, USA). One-way ANOVA was performed if there was a significant difference, and Duncan’s method was used for multiple comparisons. The values were expressed as means ± SD and differences were considered significant at *p* < 0.05.

## 3 Results

### 3.1 Screening of isolates with inhibitory activity against CP

A total of 34 isolates were screened from wild bird feces using MRS medium, and their cell-free supernatants (CFS) were tested for inhibitory activity against bird-derived CP strain using the agar well diffusion method. The results showed that isolates #7, #25, #28, and #32 exhibited strong inhibitory activity against CP (Table 1). Therefore, these 4 isolates were selected for further study. Based on 16S rRNA gene sequencing, the 16S sequences of the 4 isolates were obtained and compared with known sequences. The results revealed a 100% sequence similarity to *E. faecalis*. A phylogenetic tree was constructed based on the neighbor-joining method using MEGA 11 software, shown in Supplementary Figure S1. Thus, these 4 isolates were identified as *E. faecalis* and named YL-EF7, YL-EF25, YL-EF28, and YL-EF32.

**Table 1:** Detection of antibacterial activity of isolated bacteria against *Clostridium perfringens*.

	Inhibition zone diameter (mm)	Antibacterial activity	Item	Inhibition zone diameter (mm)	Antibacterial activity
1	14	+	18	17.5	++
2	15	++	19	16.5	++
3	16.5	++	20	16.5	++
4	17	++	21	13.5	+
5	16	++	22	14	+
6	15.5	++	23	11.5	+
7	18.5	+++	24	12	+
8	17.5	++	25	18	+++
9	17.5	++	26	16	++
10	16.5	++	27	16	++
11	16	++	28	18	+++
12	0	−	29	16	++
13	0	−	30	16.5	++
14	0	−	31	17	++
15	17	++	32	18	+++
16	0	−	33	17	++
17	17	++	34	16	++

Data are shown as mean. +, Low antibacterial activity, Antibacterial zone diameter ≤15 mm; ++, Medium antibacterial activity, 18 mm > diameter of antibacterial zone> 15 mm; +++, High antibacterial activity, Antibacterial zone diameter ≥18 mm; −, No antibacterial zone.

### 3.2 Analysis of probiotic properties

**Figure 2:**
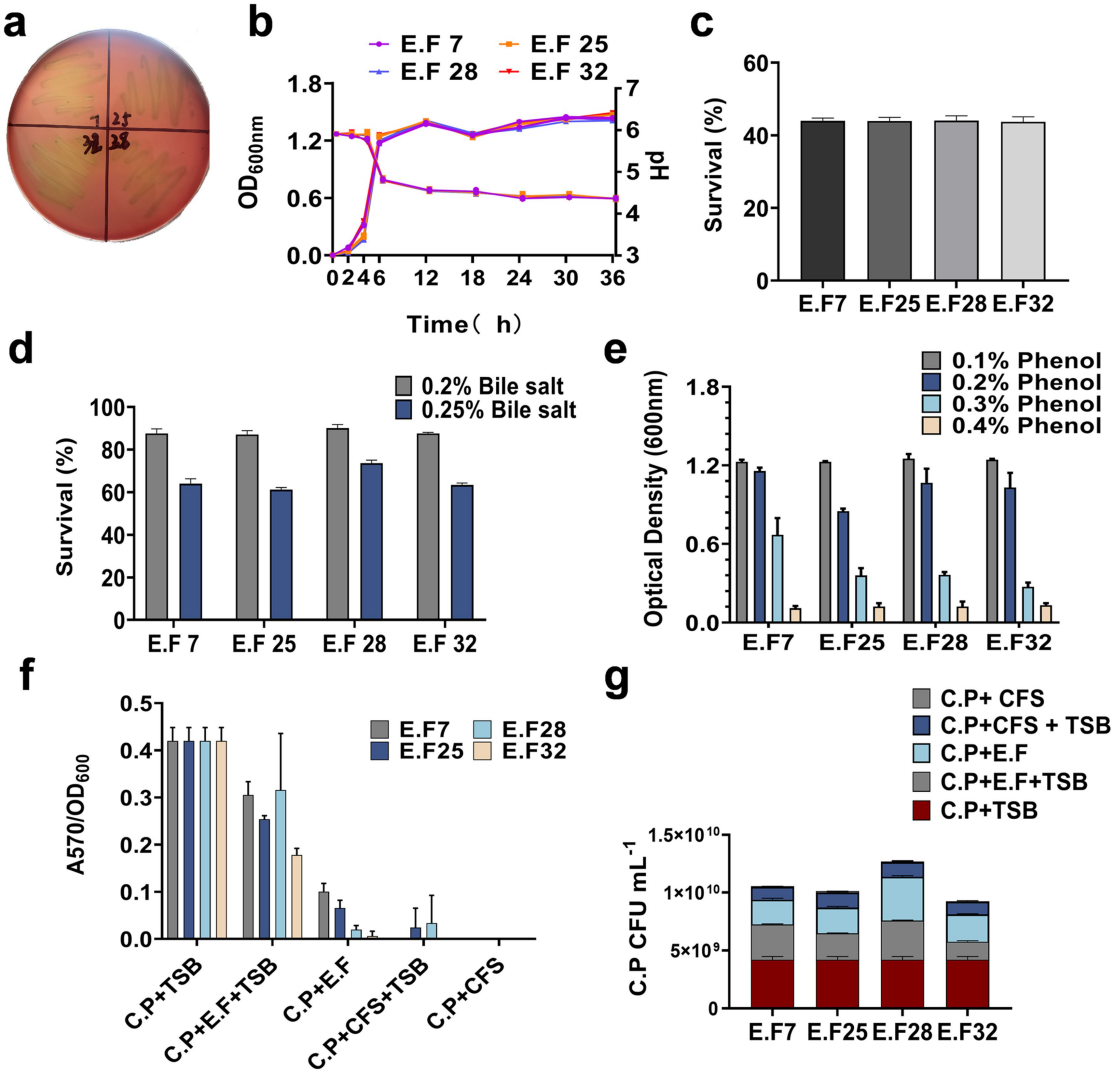
The safety and probiotic properties of *E. faecalis* strains. **(a)** Hemolytic activity of *E. faecalis* strains; **(b)** growth curves and acid production profiles of *E. faecalis* strains; The tolerance of *E. faecalis* strains to artificial gastric acid **(c)**, bile salts **(d)**, and phenol **(e)** is presented; **(f)** The inhibitory effect of *E. faecalis* strains on CP biofilm formation was assessed, **(g)** and the bacterial content of CP in the supernatants of each groups was quantified using TSN plate counts. All data are shown as mean ± standard deviation (SD).

The probiotic characteristics of the *E. faecalis* (E.F) strains were analyzed *in vitro*. The results showed that none of the strains produced hemolytic circles, indicating their safety in terms of hemolysis (Figure 2a). The growth curves and acid production capabilities of the strains in MRS medium were also evaluated (Figure 2b). When exposed to simulated gastric juice for 3 h, the strains maintained a survival rate of 40% (Figure 2c) and showed survival rates of around 90 and 65% in 0.2 and 0.25% bile salt solutions, respectively (Figure 2d). These strains can metabolize phenol, which has strong inhibitory activity, in the gastrointestinal tract. Experiments demonstrated that the *E. faecalis* strains did not show significant inhibition when cultured in MRS medium containing 0.1 and 0.2% phenol (Figure 2e). These findings indicate that the *E. faecalis* strains have strong resistance to gastric acid, high concentrations of bile salts, and phenol. They are safe, environmentally tolerant, and candidate strains for anti-CP applications.

### 3.3 Inhibition of CP biofilm formation

*Clostridium perfringens* can utilize biofilm formation as a strategy for persistent survival in various environments or hosts (23). To investigate whether the *E. faecalis* strains can inhibit CP biofilm formation, a biofilm assay was established. All CP strains were inoculated into 96-well plates, and except for the CP control group, other groups were supplemented with *E. faecalis* suspensions or CFS at ratios of 1:1 or 1:2. After 5 days of incubation, biofilm formation was observed by calculating the ratio of A570 to OD_600_. Compared with the CP control group, both the *E. faecalis* suspensions and CFS significantly reduced CP biofilm formation, especially in the EF + CFS group (Figure 2f). Additionally, the number of CP in the supernatants of each experimental group with *E. faecalis* was determined. The results showed that the presence of *E. faecalis* reduced the number of CP, particularly in the EF32 group (Figure 2g). These data indicate that the presence of the *E. faecalis* strains can effectively inhibit CP biofilm formation during the biofilm formation process, especially YL-EF25 and YL-EF32.

### 3.4 Carbon source utilization phenotype

**Figure 3:**
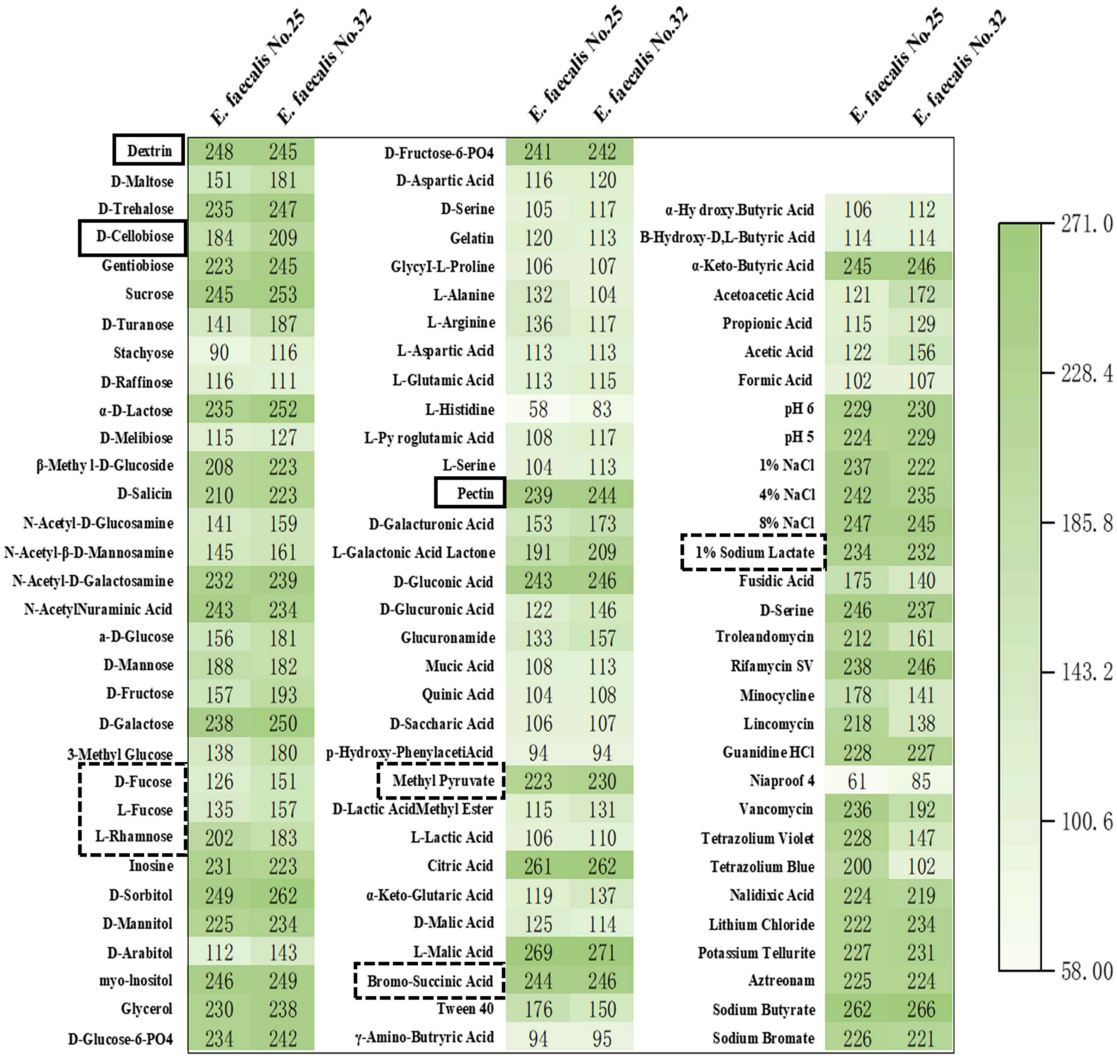
Heatmap of carbon source and chemical metabolite analysis. The metabolism of 71 carbon sources and the sensitivity to 23 chemical substances were analyzed in the *E. faecalis* strains, specifically YL-EF25 and YL-EF32. Mean≥150, Indicating strong metabolic activity toward the substance.

In this study, microbial metabolic analysis was used to investigate the metabolic capabilities of *E. faecalis* YL-EF25 and YL-EF32 toward 71 carbon sources and their sensitivity to 23 chemicals. The results showed that the two strains had similar metabolic capabilities for the 71 carbon sources and sensitivity to the 23 chemicals (Figure 3). Notably, both strains can utilize dietary fibers such as dextran, pectin, and cellobiose (Full line marker) and substrates for propionic acid synthesis pathways (Dashed line marker) as energy sources. Dietary fibers can be metabolized by probiotics to produce short-chain fatty acids (SCFA), which contribute to maintaining intestinal health.

### 3.5 Growth performance and lymphocyte proliferation

**Figure 4:**
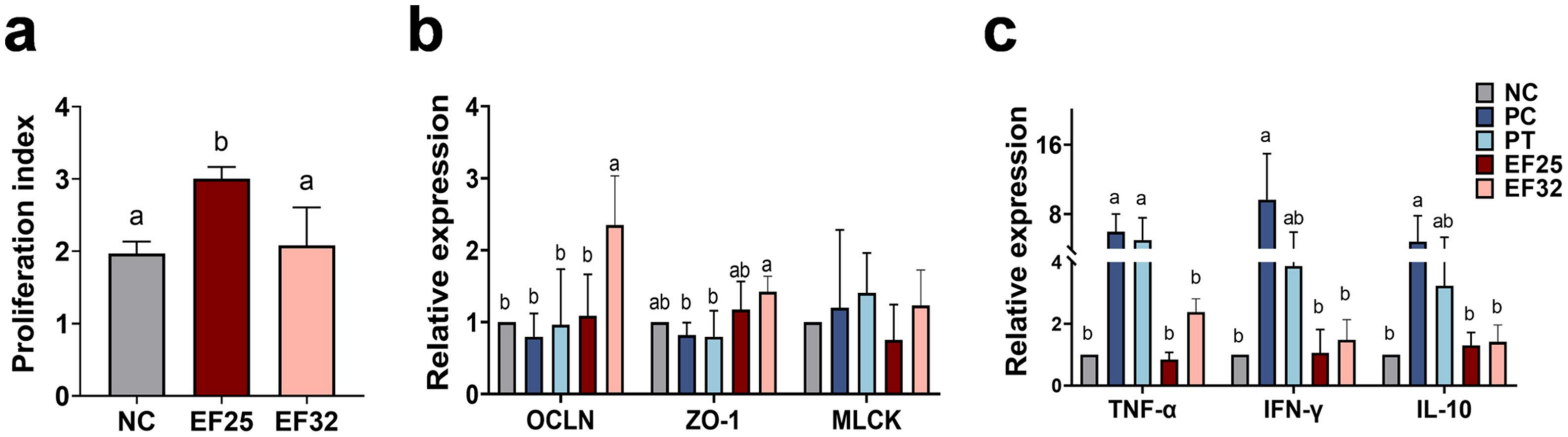
Study on the effect of *E. faecalis* YL-EF25 and YL-EF32 in preventing and controlling necrotic enteritis in broiler chickens. **(a)** The effects of *E. faecalis* YL-EF25 and YL-EF32 on the proliferative activity of peripheral blood lymphocytes in chickens, and Detection of mRNA expression levels of tight junction proteins **(b)** and inflammatory cytokines **(c)** in the jejunum of broiler chickens from each group following CP infection. Data are shown as mean ± SD. one-way ANOVA was performed. ^a,b^Means with different letters are significantly different (*p <* 0.05).

Growth performance results are presented in Table 2. Compared to the NC group, CP challenge (PC group) significantly reduced body weight gain (BWG) and increased the feed conversion ratio (FCR) in broilers during the challenge phase (14–25 days) (*p* < 0.05), but had no significant effect on feed intake (FI) (*p* > 0.05). During the challenge phase, the BWG of the PC group was significantly lower than that of the NC group, PT group, EF25 group, and EF32 group (*p* < 0.05), indicating that supplementation with YL-EF25, YL-EF32, or enramycin (PT group) partially alleviated the CP-induced reduction in weight gain. The FCR of the PC group was significantly higher than that of the NC group, PT group, EF25 group, and EF32 group (*p* < 0.05), while no significant differences in FCR were observed among the PT, EF25, and EF32 groups (*p* > 0.05). The proliferative capacity of lymphocytes is a crucial indicator for assessing immune performance. We examined the proliferative ability of peripheral blood lymphocytes in broilers raised for 14 days. Compared to the NC group, the lymphocyte proliferative capacity was significantly enhanced in the EF25 group (*p <* 0.05), while there was no significant difference in the EF32 group (Figure 4a).

**Table 2:** The 25-day broilers BW gain (BWG), feed intake (FI), and feed conversion ratio (FCR) in response to the treatments.

Treatment^1^	NC	PC	PT	EF25	EF32	SEM	*p* value
Grower (1–14 d)							
BWG (g)	47.43	49.44	49.60	49.89	51.41	2.742	0.182
FI (g)	121.72	125.48	125.13	124.23	126.98	5.014	0.316
FCR (g/g)	2.57	2.54	2.52	2.49	2.47	0.096	0.129
Challenge phase (14–25 d)							
BWG (g)	78.67^a^	61.52^c^	67.60^b^	73.88^b^	69.62^b^	4.475	0.035
FI (g)	188.76	172.87	181.84	188.4	182.4	8.117	0.147
FCR (g/g)	2.40^c^	2.81^a^	2.69^ab^	2.55^b^	2.62^b^	0.065	0.016

^a,b,c^Means with different letters are significantly different (*p* < 0.05) while with same superscripts did not have significant difference (*p* > 0.05). BWG, body weight gain; FI, feed intake; FCR, feed conversion ratio. FCR value is calculated by the ratio of FI to BWG. Data are expressed as mean±SEM, *n* = 6.

1 Treatment information: Control group: basal diet; PC group: basal diet and CP challenge; PT group: CP challenge+10 mg/kg enramycin; EF25 group: CP challenge+1 × 10^9^ CFU/kg of feed *E. faecalis* YL-EF25; EF32 group: CP challenge+1 × 10^9^ CFU/kg of feed *E. faecalis* YL-EF32.

### 3.6 Intestinal lesions and gene expression in broilers challenged with CP

Intestinal lesion scores were assessed on day 25 in the jejunum (Table 3). The intestinal macroscopic lesions are shown in Supplementary Figure S2. The CP infection group (PC) significantly increased intestinal lesion scores (*p <* 0.01). Dietary supplementation with *E. faecalis* YL-EF25 (EF25) and YL-EF32 (EF32) significantly reduced intestinal lesion scores induced by CP infection (*p <* 0.05), particularly in the EF32 group (*p <* 0.01). As shown in Figure 4b, the PC group led to a decrease in tight junction protein (TJPs)-related genes such as OCLN and ZO-1. However, the EF25 and EF32 groups increased the expression of OLCN and ZO-1 genes, with significant elevations in the EF32 group (*p <* 0.05). On the other hand, the PC and PT groups increased the expression of genes related to increased intestinal barrier permeability, such as MLCK. Dietary supplementation with *E. faecalis* YL-EF25 and YL-EF32 have a trend to alleviated the increase in MLCK, although there were no significant differences between them. This trend may indicate a modulatory role of the probiotics, but further validation with larger sample sizes or complementary functional assays is warranted. These data suggest that dietary supplementation with *E. faecalis* YL-EF25 and YL-EF32 improves intestinal barrier function. PC group and the PT group significantly increased the expression of inflammatory cytokine genes, such as TNF-α, IFN-γ, and IL-10. The EF25 and EF32 groups alleviated the upregulation of inflammatory cytokines induced by PC infection (Figure 4c). These data indicate that dietary supplementation with *E. faecalis* YL-EF25 and YL-EF32 exerts anti-inflammatory effects during CP infection.

**Table 3:** Intestinal lesion scores from animal experiment.

	Number of chickens with the indicated score						Av. score
	0	1	2	3	4	5	
NC (*n* = 8)	8	0	0	0	0	0	0
PC (*n* = 7)	2	1	1	0	3	0	2.29
PT (*n* = 9)	9	0	0	0	0	0	0^***^
EF25 (*n* = 13)	5	2	6	0	0	0	1.0^*^
EF32 (*n* = 11)	8	2	0	1	0	0	0.45^**^

^*^p < 0.005 vs. PC, ^**^p < 0.001 vs. PC, ^***^p < 0.0001 vs. PC.

### 3.7 Ileal microbiota

**Figure 5:**
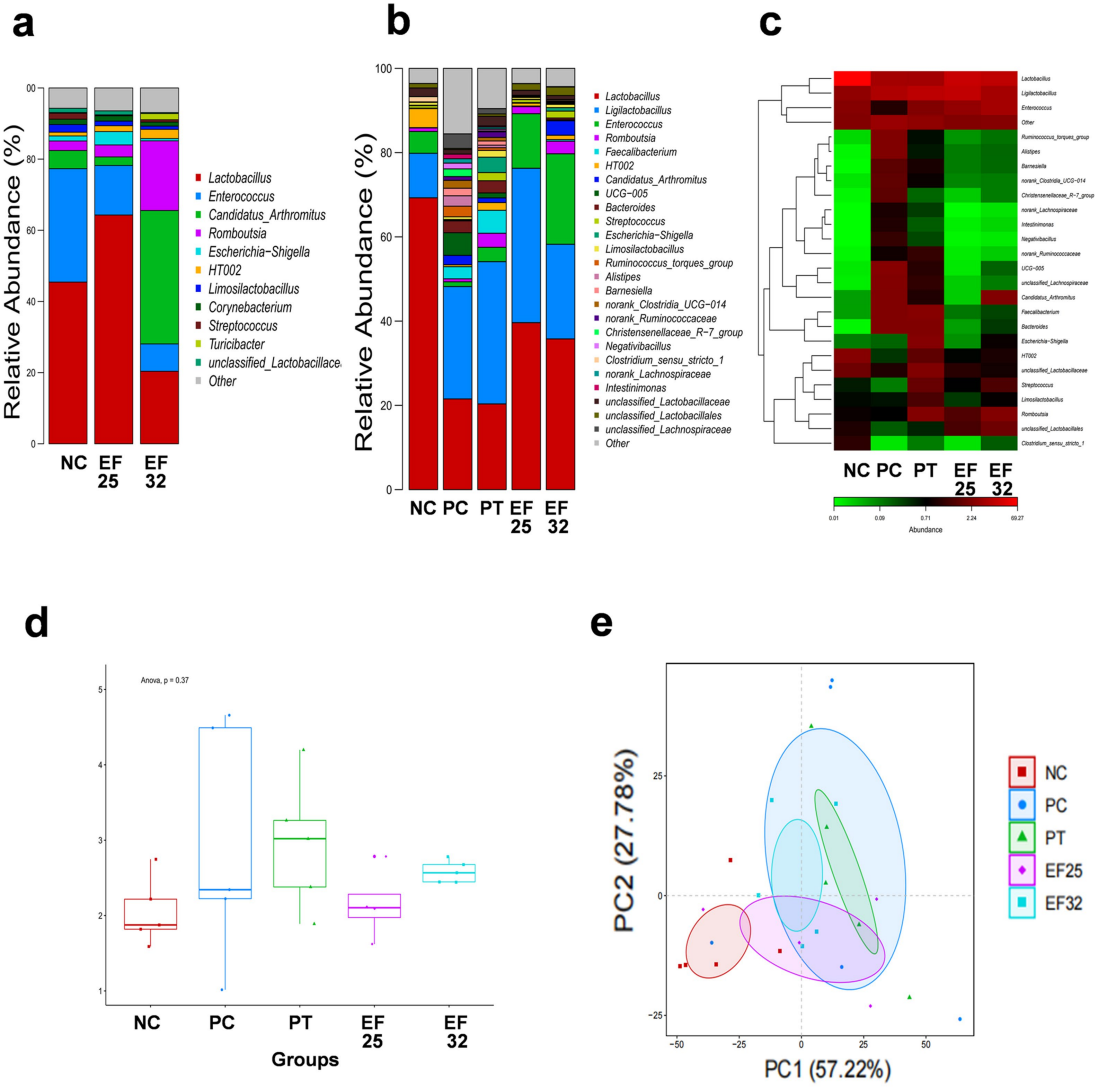
Detection of the composition and abundance of intestinal microbiota in the ileum of broiler chickens. **(a)** Barplot of intestinal microbiota at the genus level before challenge (in day 14) and **(b)** after challenge (in day 25) for each group. **(c)** Heatmap of intestinal microbiota at the genus level after challenge (in day 25) for each group. Analysis of α-diversity (Shannon index) **(d)** and β-Diversity (PCA analysis) **(e)** of intestinal microbiota after challenge (in day 25) for each group.

Through 16S rRNA sequencing analysis, we compared the composition and differences in ileal microbiota among different treatment groups on days 14 and 25. The results showed that on day 14, the EF25 and EF32 groups demonstrated distinct microbiota regulatory effects (Figure 5a). The EF25 group increased the relative abundance of *Lactobacillus*, while the EF32 group showed an increased relative abundance of segmented filamentous bacteria (SPF) in the ileal microbiota of chickens. On day 25, compared to the normal group (NC), the necrotic enteritis group (PC) and the antibiotic group (PT) exhibited a decrease in Lactobacillus abundance in the ileal microbiota and a certain degree of microbiota disruption. However, the EF25 and EF32 groups alleviated the microbiota disruption caused by necrotic enteritis in chickens. Compared to the PC group, the EF25 and EF32 groups had higher Lactobacillus abundance (Figure 5b), and their microbiota abundance and composition were more similar to the NC group (Figure 5c). Further comparison of the Shannon index and principal component analysis (PCA) among groups further demonstrated that the microbiota abundance and composition structure of the EF25 and EF32 groups were closer to those of the NC group (Figures 5d,e).

## 4 Discussion

The application of antibiotics in the animal husbandry sector has been progressively restricted, and the hazards associated with bacterial diseases that were previously controlled by antibiotics have become increasingly severe. Acute infections caused by CP can lead to a decline in animal production performance, disruption of intestinal microbiota balance, and the induction of severe intestinal inflammatory responses. A plethora of evidence suggests that probiotics can effectively alleviate the symptoms of necrotic enteritis (NE) in chickens and improve overall animal health (9). A key attribute of probiotics lies in their capacity to synthesize short-chain fatty acids (SCFAs), which primarily include acetic acid, propionic acid, and butyric acid, and are recognized for their ability to suppress pathogenic bacterial colonization, mitigate inflammatory responses, and modulate intestinal immune homeostasis (28). *E. faecalis* sp. have been reported to produce long-chain fatty acids and SCFAs such as propionic acid, exhibiting favorable effects on cholesterol reduction and weight management (29). There are three known pathways for propionic acid synthesis, the succinate pathway utilizing succinic acid, the acrylate pathway utilizing lactic acid, and the propanediol pathway utilizing fucose or rhamnose. Dietary fiber, such as dextran and pectin, serves as an energy source for probiotics to produce SCFAs (30). Carbon source metabolism analysis revealed that *E. faecalis* YL-EF25 and YL-EF32 exhibit metabolic activity toward intermediate substrates (Dashed line marker) in the three propionic acid synthesis pathways and dietary fibers such as dextran, pectin, and cellobiose (Full line marker), indicating their potential to exert intestinal protective effects through the production of SCFAs.

Tight junctions serve as a crucial structural foundation for the intestinal mucosal barrier, playing a pivotal role in selectively regulating intestinal permeability and preventing the entry of harmful substances. The primary tight junction proteins include ZO-1 and Occludin (31). In this study, necrotic enteritis (NE) was found to decrease the expression levels of ZO-1 and Occludin in the jejunum, resulting in increased intestinal permeability. Conversely, *E. faecalis* strains YL-EF25 and YL-EF32 were observed to upregulate the expression of ZO-1 and Occludin in the jejunum. Similar findings have been reported, where *Bacillus subtilis* HW2 was also shown to increase the expression of ZO-1 and Occludin following CP challenge (32). Furthermore, our study revealed that *E. faecalis* YL-EF32 exhibited a more pronounced effect in improving intestinal barrier function compared to *E. faecalis* YL-EF25, which may account for the lower intestinal lesion scores observed in the EF32 group.

Inflammatory cytokines play a communicative role in regulating inflammatory responses. Previous research has demonstrated that probiotics can ameliorate CP-induced inflammatory responses by modulating inflammatory cytokines (16, 33). Our results indicated that *E. faecalis* YL-EF25 and YL-EF32 were able to reduce the elevated expression levels of TNF-α, IFN-γ, and IL-10 in the jejunum caused by NE. Similarly, a prior study found that *Clostridium butyricum* could decrease the increased expression levels of TNF-α and IL-10 in the jejunum induced by NE (34). Although the antibiotic group resisted intestinal damage from NE, it exhibited an elevated inflammatory response, which may be attributed to antibiotic-induced gut microbiota disruption.

The gut microbiota is a key component in maintaining intestinal health, exerting positive effects on the regulation of intestinal inflammation and the protection of the intestinal barrier. Probiotics play a significant role in modulating the composition of the gut microbiota. Previous studies have shown that *E. faecalis* can counteract intestinal pathogens such as *Listeria monocytogenes* and pathogenic *Bacillus* species by producing lactic acid and bacteriocins (35, 36). SPF has the ability to penetrate the mucosal barrier, promoting the secretion of IL-17A by Th17 cells and IL-22 by ILC3, thereby defending against the invasion of extraintestinal pathogens (37). Our study results revealed that *E. faecalis* YL-EF25 and YL-EF32 increased the relative abundance of *Lactobacillus* and *Candidatus Arthromitus* (SPF) in the ileal microbiota, respectively. The ileal microbiota of the NE and antibiotic groups exhibited gut microbiota disruption. However, *E. faecalis* YL-EF25 and YL-EF32 were able to regulate the NE-induced microbiota disruption, restoring the gut microbiota structure and abundance toward that of the control group. These findings may explain the reason for enhanced barrier function and reduced lesion scores observed in the groups treated with these probiotics. In addition, both strains of *E. faecalis* demonstrated promising regulatory effects on locally-sourced broiler growth performance, but the FCR value (2.49 ± 0.11) of broilers in this study was higher than the typical range of commercial broilers (1.5–1.8). It may be influenced by impure breed of broiler from local farm. To address this limitation, future research will use certified commercial broiler to more clearly evaluate NE prevention strategies.

## 5 Conclusion

*Enterococcus faecalis* strains YL-EF25 and YL-EF32 derived from wild bird exhibit inhibitory effects on the growth of CP. These strains are capable of utilizing dietary fiber and propionate as substrates for microbial synthesis and demonstrate tolerance to inhibitory substances such as gastric acid, bile salts, and phenol. Our vivo test implying that addition of *E. faecalis* YL-EF25 and YL-EF32 improved growth performance and mitigated NE-induced gut injury in broilers by regulating intestinal barrier function, intestinal mucosal inflammatory responses, and the gut microbiota. We intend to further investigate whether *E. faecalis* YL-EF25 and YL-EF32 supplementation throughout the experimental period is a successful alternative for controlling CP infection in broilers.

## Data Availability

The raw data supporting the conclusions of this manuscript will be made available by the authors, without undue reservation, to any qualified researcher. The data presented in the study are deposited in the Sequence Read Archive (SRA) repository, accession number SRR33542538.

## Footnotes

^1^https://blast.ncbi.nlm.nih.gov/Blast.cgi, accessed on 19 June 2023.
