# Supplementary material for: Enterococcus faecalis strains derived from wild bird provide protection against Clostridium perfringens challenge in locally-sourced broilers
Source: Front Vet Sci. 2025 May 15;12:1601605. doi: 10.3389/fvets.2025.1601605 (PMC12121489; doi:10.3389/fvets.2025.1601605)
Supplement: Supplementary file 1 [file Table_1.docx]

***Enterococcus faecalis* strains derived from wild bird provide protection against *Clostridium perfringens* challenge in broiler chickens**

Supplementary Material

**Supplementary Table**

**Supplementary Table S1 The nutrient composition of the feed for experimental animals.**

| Ingredienta | Diet (%) |
| --- | --- |
| Ground yellow corn  Soybean meal  Soybean oil  Ground limestone  Dicalcium phosphate  Salt  Choline chloride  DL-Met  Micronutrients  **Calculated nutrients level (%)**  ME (kcal kg−1)  Crude Protein  Crude Fiber  Digestible lysine  Digestible methionine  Digestible threonine  Available phosphorus  Calcium  NaCl | 56.0  37.0  3.66  0.57  1.78  0.30  0.10  0.25  0.34  3100  22.0  5.0  0.8  0.4  0.65  0.45  0.6  0.37 |

Abbreviation: ME, Metabolizable energy.

Vitamin premix per kg contains the following: Vitamin A, 10,000 IU; Vitamin D₃, 2,500 IU; Vitamin E, 15 IU; Vitamin K₃, 2.5 mg; Vitamin B₁, 1.5 mg; Vitamin B₂, 6 mg; Pantothenic acid, 11 mg; Niacin, 40 mg; Vitamin B₆, 3.5 mg; Biotin, 0.15 mg; Folic acid, 1.2 mg; Vitamin B₁₂, 12 μg.

Mineral premix per kg contains the following: Calcium, 10 g; Total phosphorus, 7 g; Available phosphorus, 4.5 g; Sodium, 2 g; Chloride, 2 g; Iron, 90 mg; Zinc, 70 mg; Manganese, 65 mg; Copper, 9 mg; Iodine, 0.35 mg; Selenium, 0.18 mg.

**Supplementary Table S2 Primer for q-RT PCR**

| Primers | Sequences（5’→3’） |
| --- | --- |
| Ch-OLCN | F: cctctgccacatcctggtat  R: gctgagatggacagcatcaa |
| Ch-ZO-1 | F: ccgaagcattccatcttcat  R: ggtcagccagatgtggattt |
| Ch-MLCK | F: TTGACATGGAGGTTGTGGAA  R: GAAGTGACGGGACTCCTTGA |
| Ch-TNF-α | F: CCCCTACCCTGTCCCACAA  R: TGAGTACTGCGGAGGGTTCAT |
| Ch-IFN-γ | F: CTTCCTGATGGCGTGAAGA  R: GAGGATCCACCAGCTTCTGT |
| Ch-IL-10 | F: CAGCACCAGTCATCAGCAGAGC  R: GCAGGTGAAGAAGCGGTGACAG |
| Ch-GAPDH | F: TGCTGCCCAGAACATCATCC  R: ACGGCAGGTCAGGTCAACAA |

**Supplementary Figure**

**
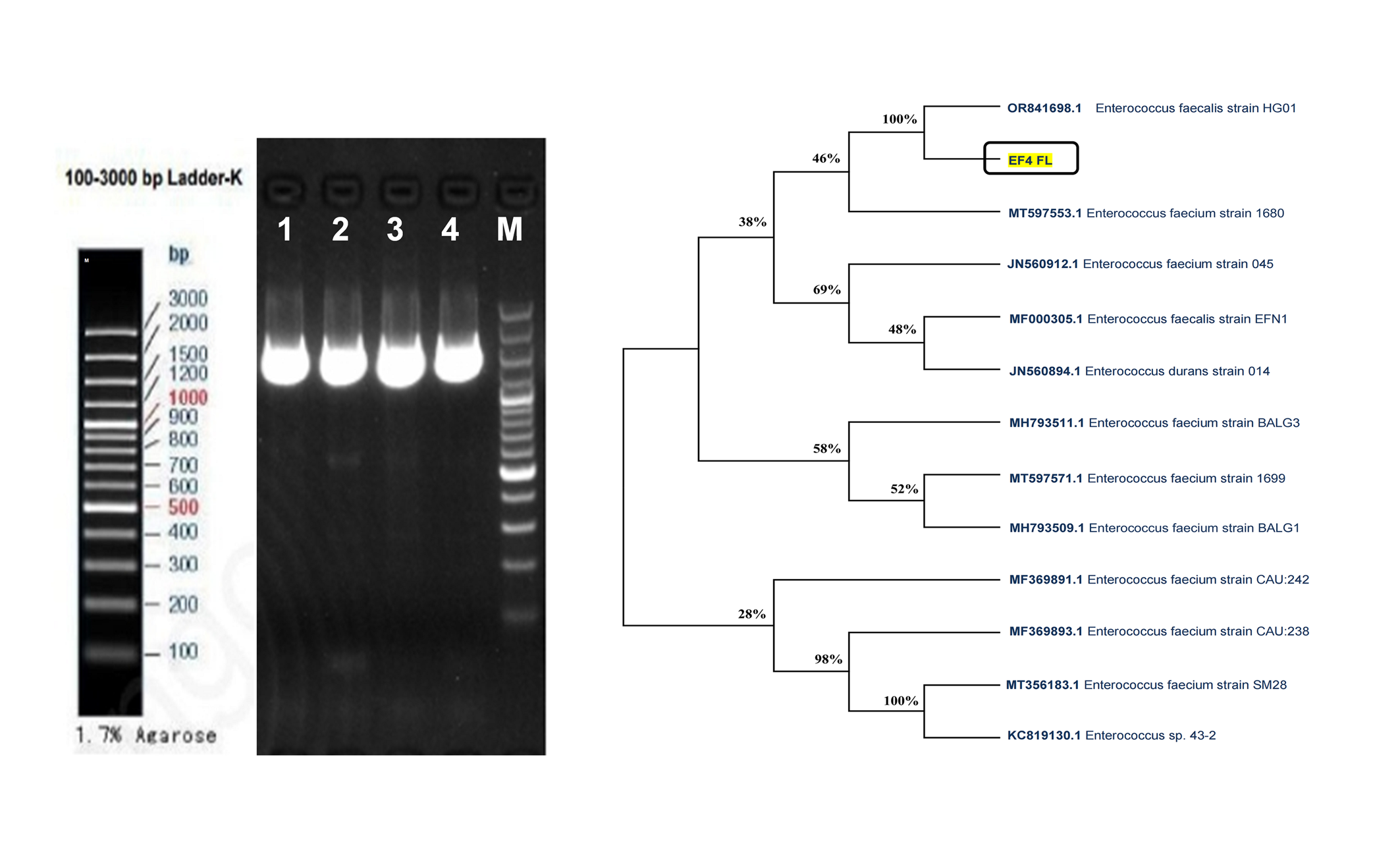
**

**Fig.S1 16S rRNA identification and phylogenetic tree analysis of *E. faecalis* strains** Polygenetic tree showing the relative positions of the 4 strains isolated from the wild fece and their related species. The tree was constructed using the neighbor-joining method based on approximately 1500 bp of 16S rDNA sequences. Bootstrap values are shown at the branching points. (Yellow highlighted) means strains identified in the present study.

**
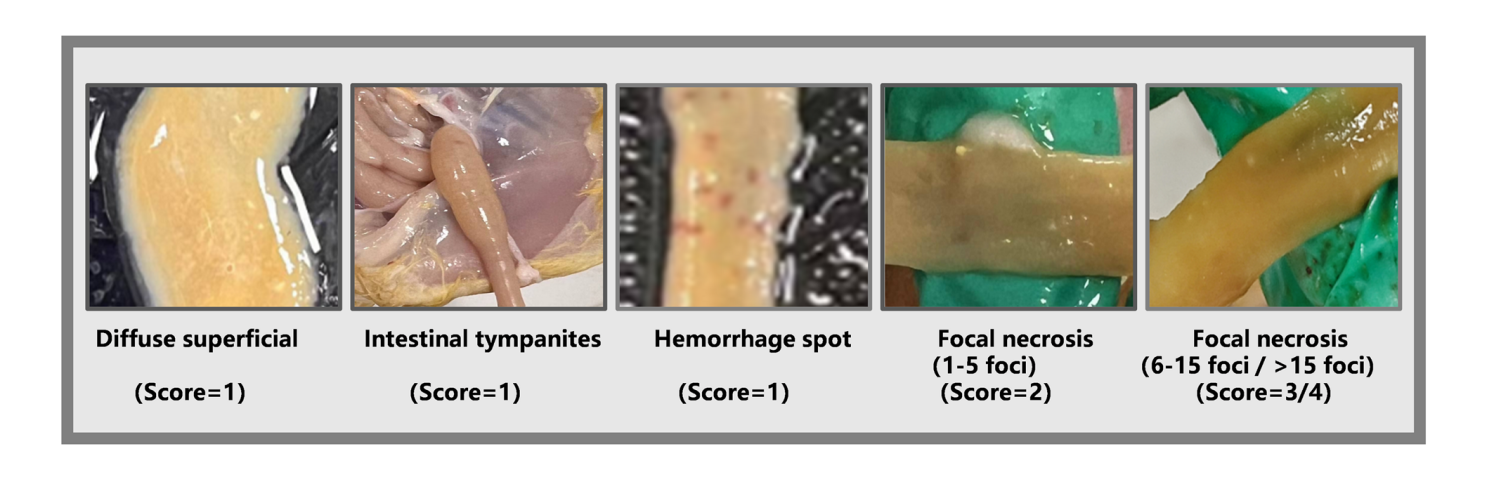
**

**Fig.S2 Schematic diagram of the scoring standard for chicken necrotic enteritis lesions** Intestinal lesions were scored as follows: 0 = no gross lesions; 1 = thin or friable wall or hemorrhage spot; 2 = focal necrosis or ulceration, or non-removable fibrin deposit, 1 to 5 foci; 3 = focal necrosis or ulceration, or non-removable fibrin deposit, 6 to 15 foci; 4 = focal necrosis or ulceration, or non-removable fibrin deposit, 16 or more foci; 5 = patches of necrosis 2 to 3 cm long.
